# Unintended Perinatal Health Consequences Associated With a Swedish Family Policy

**DOI:** 10.1001/jamapediatrics.2024.0378

**Published:** 2024-04-08

**Authors:** Enrico Debiasi, Helena Honkaniemi, Siddartha Aradhya, Anders Hjern, Ann-Zofie Duvander, Sol P. Juárez

**Affiliations:** 1 Centre for Health Equity Studies, Stockholm University/Karolinska Institutet, Stockholm, Sweden; 2 Department of Public Health Sciences, Stockholm University, Stockholm, Sweden; 3 Stockholm University Demographic Unit, Department of Sociology, Stockholm University, Stockholm, Sweden; 4 Department of Humanities and Social Sciences, Mid Sweden University, Östersund, Sweden

## Abstract

**Importance:**

The 1980 and 1986 Swedish so-called speed premium policies aimed at protecting parents’ income-based parental leave benefits for birth intervals shorter than 24 and 30 months, respectively, but indirectly encouraged shorter birth spacing and childbearing at older ages, both risk factors for several perinatal health outcomes. Whether those policy changes are associated with perinatal health remains unknown.

**Objective:**

To evaluate the association between the 1980 and 1986 speed premium policies and perinatal health outcomes.

**Design, Setting, and Participants:**

This cross-sectional study investigated data from 1 762 784 singleton births in the Swedish Medical Birth Register from January 1, 1974, through December 31, 1991. Data were analyzed from October 11, 2022, to December 12, 2023.

**Interventions:**

Speed premium policy introduction (January 1, 1980) and extension (January 1, 1986).

**Main Outcomes and Measures:**

Total population register data were used in an interrupted time series analysis with segmented logistic regression to calculate the odds of preterm birth, low birth weight, small for gestational age (SGA) at preterm, and stillbirth measured before and after the speed premium policy reforms. Subgroup analyses by maternal origin were conducted to evaluate changes by different policy responses.

**Results:**

Among 1 762 784 births analyzed, 4.8% were preterm (of which 12.0% were SGA), 3.2% had low birth weight, and 0.3% were stillbirths. The 1980 speed premium policy was associated with a 0.3% monthly increase in the odds of preterm birth compared with the period before the reform (odds ratio [OR], 1.0029 [95% CI, 1.002-1.004]), equivalent to a 26.4% increase from January 1, 1980, to December 31, 1985. After the 1986 relaxation of the policy, preterm birth odds decreased 0.5% per month (OR, 0.9951 [95% CI, 0.994-0.996]), equivalent to an 11.1% decrease across the next 6 years. Low birth weight displayed a similar pattern for both reform periods, that is, increased 0.2% (OR, 1.0021; 95% CI, 1.001-1.003) per month in 1980 through 1985 compared with baseline, and decreased 0.3% (OR, 0.9975; 95% CI, 0.996-0.998) per month in the following period, but was attenuated when considering low birth weight at term. Odds of SGA at preterm were decreased after 1980 (OR, 0.9965; 95% CI, 0.994-0.999) but not in 1986 (OR, 1.0009; 95% CI, 0.998-1.003), whereas stillbirths did not change following either reform (1980: OR, 1.0020 [95% CI, 0.999-1.005]; 1986: OR, 1.0002 [95% CI, 0.997-1.003]). Subgroup analyses suggested that perinatal health changes were restricted to births to Swedish- and Nordic-born mothers, the primary groups to adjust their fertility behaviors to the reforms.

**Conclusions and Relevance:**

Despite its economic advantages for couples, especially for mothers, the introduction of the speed premium policy was associated with adverse perinatal health consequences, particularly for preterm births. Family policies should be carefully designed with a “Health in All Policies” lens to avoid possible unintended repercussions for fertility behaviors and, in turn, perinatal health.

## Key Points

**Question:**

What is the association of a Swedish family policy protecting parental leave payment levels in short birth intervals with perinatal health outcomes?

**Findings:**

In this cross-sectional study using an interrupted time series analysis of 1 762 784 births in Sweden, stricter birth spacing requirements for protected parental leave benefits were associated with significantly increased odds of preterm births and low birth weight but not of small for gestational age at preterm or stillbirths. Subsequently relaxed birth spacing requirements were associated with decreased odds of preterm births and low birth weight.

**Meaning:**

Family policies aiming to provide socioeconomic protections may motivate changes in fertility behaviors with negative consequences for perinatal health.

## Teaser

This cross-sectional study uses an interrupted time series analysis to assess whether an association exists between Swedish speed premium family policies protecting parental leave payment levels in short birth intervals and perinatal health outcomes among infants.

## Introduction

Family policies worldwide have been associated with changes in fertility behaviors, with repercussions for perinatal health.^1^ In Sweden, decades of family policy reform not only have increased overall fertility rates but also have altered the temporal patterning of fertility, by shortening birth spacing and increasing the average maternal age at first birth.^2,3^ Both short birth spacing and advanced maternal age (ie, older than 40 years) are known risk factors for severe outcomes, including preterm birth, low birth weight (LBW), small for gestational age (SGA), and stillbirth.^4,5,6^ However, little has been done to investigate specific instances of family policy linked to fertility changes and their associations with perinatal health.

One relevant policy in Sweden, commonly referred to as the speed premium, aims to protect the economic stability of parents who have children in “quick” succession. Before 1980, parents (especially mothers) who had children within short intervals may have received lower parental leave benefits for the second child due to a decreased salary, that is, if they reduced their working hours and did not return to full-time work between births. With the 1980 speed premium, parents became eligible to maintain the same benefits for children born within 24 months of each other. In 1986, this window was extended to 30 months. Table 1, following the revised Template for Intervention Description and Replication reporting guideline for population health and policy interventions, gives a more detailed overview of the speed premium.^7,8^

**Table 1.** Overview of the Speed Premium Policy Based on the Revised Template for Intervention Description and Replication Checklist for Population Health and Policy Interventions

Brief name	Speed premium (Swedish: snabbhetspremien)
Why	Parental leave allowances are calculated based on each parent’s work-related earnings prior to a live birth. Parents are granted payouts as a percentage of the average salary they earned in the 8 mo prior to birth (with a cost ceiling) when going on leave after having a child. Following the introduction of the parental leave system in 1974, benefits were reimbursed at 90% of the salary, then decreased to 80% in the 1990s following the economic recession, and thereafter to 77.6% in the 2000s, which is in effect today. Individuals with no income or below a determined threshold in the 8 mo prior to birth receive a flat-rate allowance (ie, the basic level). After a parental leave period for 1 child, parents need to work at least 8 mo with an average salary higher than the threshold to be eligible for earnings-related benefits for the subsequent child. Parents who give birth to children in quick succession and are not able to return to work in between births or do so only part-time (given that parents are entitled to reduce their working hours to 80% of full-time) may see their benefits calculated on the basis of a lower income. The speed premium reform is thus intended to protect the income-related parental leave benefits of parents who have children in quick succession.
What materials	The nature of benefits provided by the intervention is monetary, in the form of a higher parental leave allowance for eligible parents. The value of benefits provided by the intervention can be understood by examining the difference between the basic flat-rate and the maximum income-based parental leave allowance (ie, an indication of the potential size of the speed premium). In January 1980, the basic-level allowance was 37 SEK/d (approximately US $3.50/d), while the maximum parental leave allowance was 257 SEK/d (approximately US $24.25/d). In 2022, the basic-level allowance was 250 SEK/d (approximately US $23.60/d) while the maximum allowance was 1027 SEK/d (US $97.00/d). The monetary return of the speed premium can thus be substantial, given that a relatively high percentage of parents work and are eligible for parental leave benefits higher than the basic flat rate (in 2021, 10% of eligible women and 3% of men received parental leave benefits at the basic level; for Swedish-born parents, this proportion was even lower, at 2.7% of women and 0.6% of men).^7^
What and how	Since 1980, the speed premium allowed parents to maintain the parental leave benefits that they had for their first child if they had the second child within an interval of 24 mo between live births. In 1986, the eligibility interval was extended to 30 mo. Benefits are delivered by automatically renewing the parental leave allowance of the previous child for the subsequent child.
Who provided	The Swedish Social Insurance Agency (Swedish: Försäkringskassan).
Where	Nationwide intervention in Sweden for eligible parents.
When, how often, and variation over time	1974: The speed premium existed as a legal practice provided that the interval between births did not exceed 12 mo, yet few parents could take advantage of it. 1980: The speed premium became a statutory codified law, and eligibility was extended up to 24 mo, after which more parents found it manageable to take advantage of the law. 1986: Speed premium eligibility was extended to 30-mo birth intervals. 2024: The speed premium is still in place.
How well	The delivery of the intervention was faithful to its expectations, with previous studies indicating how fertility behaviors adapted to the policy thresholds. However, the policy did not intend for its target population to adjust its fertility behaviors, but rather to provide protection for parents who already met its criteria for short birth spacing.

**Figure 1.**
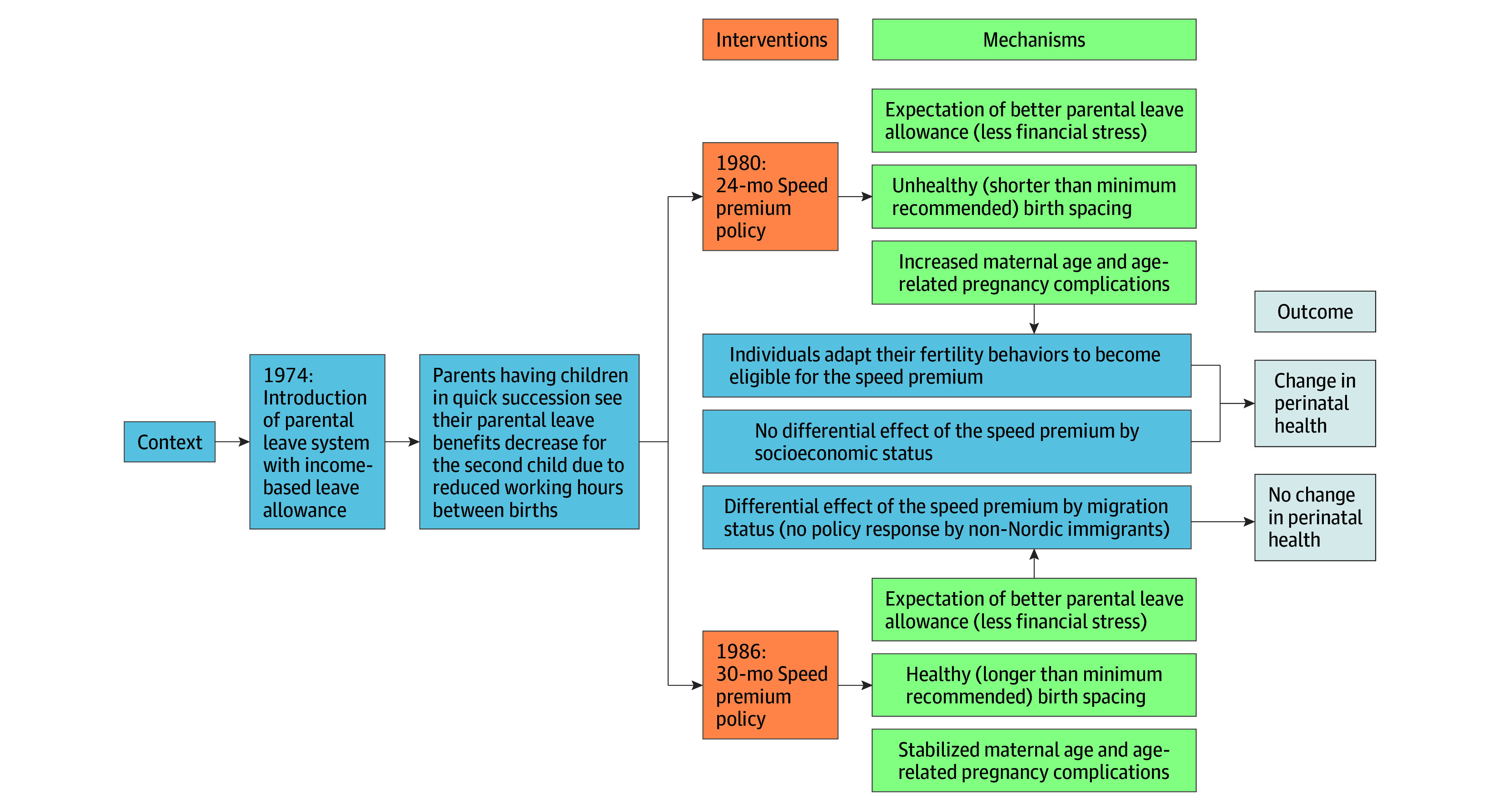
Logic Model of Change in Perinatal Health

The 1980 speed premium has previously been associated with a significant reduction in birth spacing, with a greater proportion of parents having children 18 to 24 months apart.^9^ Yet the World Health Organization today recommends that parents wait at least 24 months after a live birth before conceiving again to avoid adverse health consequences for the mother and the child.^7^ The speed premium may have also encouraged childbearing among older women intending to have 2 or more children, given that its income protection incentive was especially advantageous for parents at more advanced stages of their careers, that is, with higher income-based rather than flat-rate benefits.^2,9^ The 1986 relaxation of the speed premium criterion to 30-month intervals appeared to increase the proportion of parents having children between 24 and 30 months apart.^2,9,10^ In light of a general preference for having 2 or more children in Sweden,^11^ the policy relaxation provided an opportunity to have more than 1 child within a longer time frame, thus encouraging first childbearing earlier in the reproductive life span. Through these changed fertility behaviors, the reforms could have also affected perinatal health risks, including preterm birth, LBW, SGA, and stillbirth. Figure 1 provides a logic model of change, summarizing possible mechanisms for health changes. Based on the aforementioned reasoning, we applied an interrupted time series (ITS) approach to evaluate the association between the speed premium reforms and preterm births, LBW, SGA, and stillbirths in Sweden.

## Methods

This cross-sectional study, conducted as part of the Unintended health consequences of Swedish parental leave policy (ParLeHealth) research project, was conducted based on a published peer-reviewed protocol.^12^ This study followed the Strengthening the Reporting of Observational Studies in Epidemiology (STROBE) reporting guideline. The study was approved by the Swedish Ethical Review Authority. The authority waived the need for obtaining informed consent because the data used for analyses were pseudonymized.

### Data

**Figure 2.**
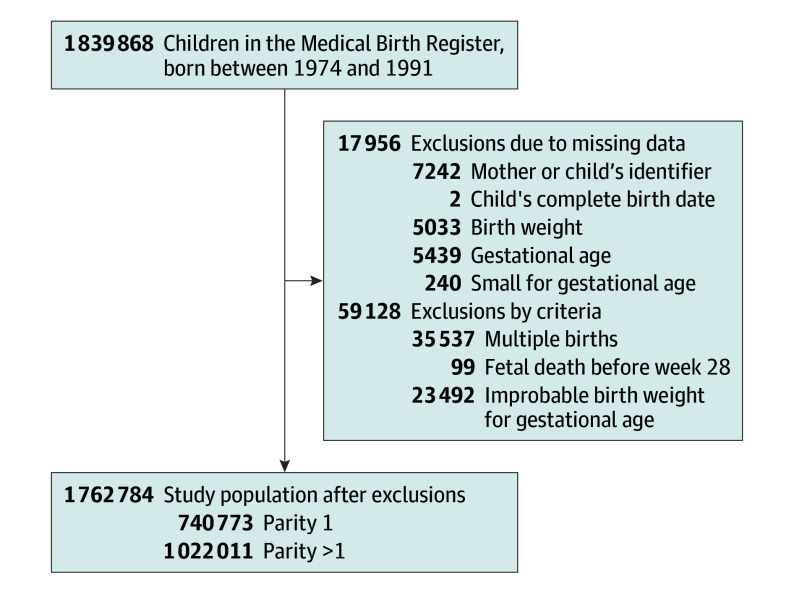
Flowchart of Study Population

Information was drawn from the Medical Birth Register, which has captured approximately 99% of all births in Sweden since 1973.^13^ This information included data on children’s and mothers’ health at birth. Among 1 839 868 births from 1974 to 1991, we excluded data with missing maternal information or incomplete birth dates (Figure 2). After also excluding multiple births, fetal deaths before 28 0/7 weeks were dropped to comply with the official definition of stillbirth valid for the studied period (fetal deaths before this week were not reported systematically). We further excluded observations with missing information on any outcome of interest, or with biologically implausible birth weight for gestational age based on national references.^14,15^

### Outcome Variables

*Preterm birth* was defined as a birth before 37 completed weeks of gestation (ie, 37 0/7 weeks or <259 gestational days). In the Medical Birth Register, gestational age is calculated using the best available information. The register primarily considers ultrasonography-based estimates, unless large discrepancies exist with the date of the last menstrual period reported by the mother, and on clinical assessment by the delivery unit, in which case the estimate is based on hierarchical consideration of the available information.^16,17^

We defined *LBW* as weight below 2500 g, independent of gestational age. We also considered LBW *at term,* defined as less than 2500 g after 37 0/7 weeks. We defined *SGA* as birth weight 2 SDs below the mean by gestational age and sex, based on the appropriate Swedish intrauterine growth curve.^14^ Our analyses consider SGA *at preterm*, as previous research suggests that it is a more accurate measure for pathologically growth-restricted fetuses (vs constitutionally small fetuses) and therefore for perinatal health.^18^ *Stillbirth* was defined as fetal death after 28 0/7 weeks’ gestation. We included a descriptive analysis of maternal diabetes (including pregestational and gestational diabetes, as per the *International Classification of Diseases, Eighth Revision*) as an indicator of an age-related pregnancy condition.^19^

### Statistical Analysis

We used an ITS design with multiple treatment periods.^20,21^ Based on the 2 reform dates (January 1, 1980, and January 1, 1986), the analyses were divided into 3 periods spanning a total of 6 years (January 1, 1974, through December 31, 1979; January 1, 1980, through December 31, 1985; and January 1, 1986, through December 31, 1991). The first period covered the years during which the speed premium was not yet statutory law and income protection was granted only for 12 months’ spacing between births. The second period captured years in which birth spacing lower than 24 months was linked to more advantageous parental leave benefits. In the third period, the maximum birth spacing for eligibility of the speed premium was extended to 30 months.

The objective of an ITS is to estimate changes in the level and trend of an outcome following an interruption (eg, a policy) by extrapolating the development of the outcome after the interruption and comparing this counterfactual scenario to any observed changes.^21,22^ We thus conducted an ITS with segmented logistic regression to model the odds of the outcomes over time. The regression coefficients reflected the mean percentage change in the odds of each outcome from one month to another. In addition to a crude model, we adjusted for birth month to account for seasonality and minimize autocorrelation.^21,22^

Sensitivity analyses and robustness checks were also performed. First, we stratified analyses by immigrant status based on the mother’s country or region of origin, given that non-Nordic immigrants in Sweden were previously shown to have little to no response to the policy.^10^ Mothers were thus categorized into 1 of 5 groups: Swedish (ie, native born), Nordic (excluding Sweden), Western (27 European Union countries, US, Canada, and Oceania), Eastern European (Poland, former Soviet Union, former Yugoslavia, and European countries outside the 27 European Union countries) and Non-Western (Central and South America, Africa, Middle East, and Asia). Since we did not expect to observe any changes in their outcomes, mothers born outside Nordic countries were treated as a pseudocontrol group for our analyses.

Next, to explore the association of birth spacing with adverse perinatal health outcomes, we divided the analyses by parity, considering first births separately from higher-order births. This was to ascertain the degree to which birth spacing alone could be associated with perinatal health outcomes, given that the first-born subsample was not exposed to a reduction in birth spacing. To more explicitly examine the role of advanced maternal age, we plotted both maternal age at childbirth and rate of maternal diabetes.

We evaluated the possibility that results for preterm birth were associated with changes in the method used for estimating gestational age, namely the shift from last menstrual period to ultrasonography-based estimations that occurred during the study years.^23^ We additionally excluded children born before 29 weeks’ gestation to evaluate whether variations in preterm birth odds were associated with increased survival of extremely preterm children. Finally, we replicated the main analyses using another common ITS approach based on linear models of aggregated data (eMethods in Supplement 1).^24^

All statistical analyses were conducted using Stata, version 16.1 (StataCorp). Statistical significance was defined as a 95% CI excluding 1. Data were analyzed from October 11, 2022, to December 12, 2023.

## Results

In the analyzed sample of 1 762 784 births, 4.8% were preterm (of which 12.0% were SGA), 3.2% had LBW, and 0.3% were stillbirths. Further descriptive characteristics by period are presented in Table 2.

**Table 2.** Descriptive Statistics of 1 762 784 Births Across 3 Analyzed Time Periods

Characteristic	Births, %		
	January 1, 1974, to December 31, 1979 (n = 574 146)	January 1, 1980, to December 31, 1985 (n = 538 237)	January 1, 1986, to December 31, 1991 (n = 650 401)
Outcome			
Preterm	4.3	5.0	5.1
LBW	3.0	3.2	3.3
SGA at preterm	12.3	11.8	11.7
Stillbirths	0.4	0.3	0.3
Maternal age at birth, y			
<30	73.9	65.7	63.5
30-39	25.2	33.0	34.7
≥40	0.9	1.3	1.8
Mean (SD), y	26.5 (4.9)	27.6 (5.1)	28.0 (5.1)
Parity			
First order	42.8	40.8	42.3
Higher order	57.2	59.2	57.7
Country/region of birth			
Sweden	88.7	87.9	87.5
Nordic	6.6	6.2	4.6
Western	1.4	1.3	1.1
Eastern Europe	1.9	1.9	1.7
Non-Western	6.6	2.8	5.1

Abbreviations: LBW, low birth weight; SGA, small for gestational age.

**Figure 3.**
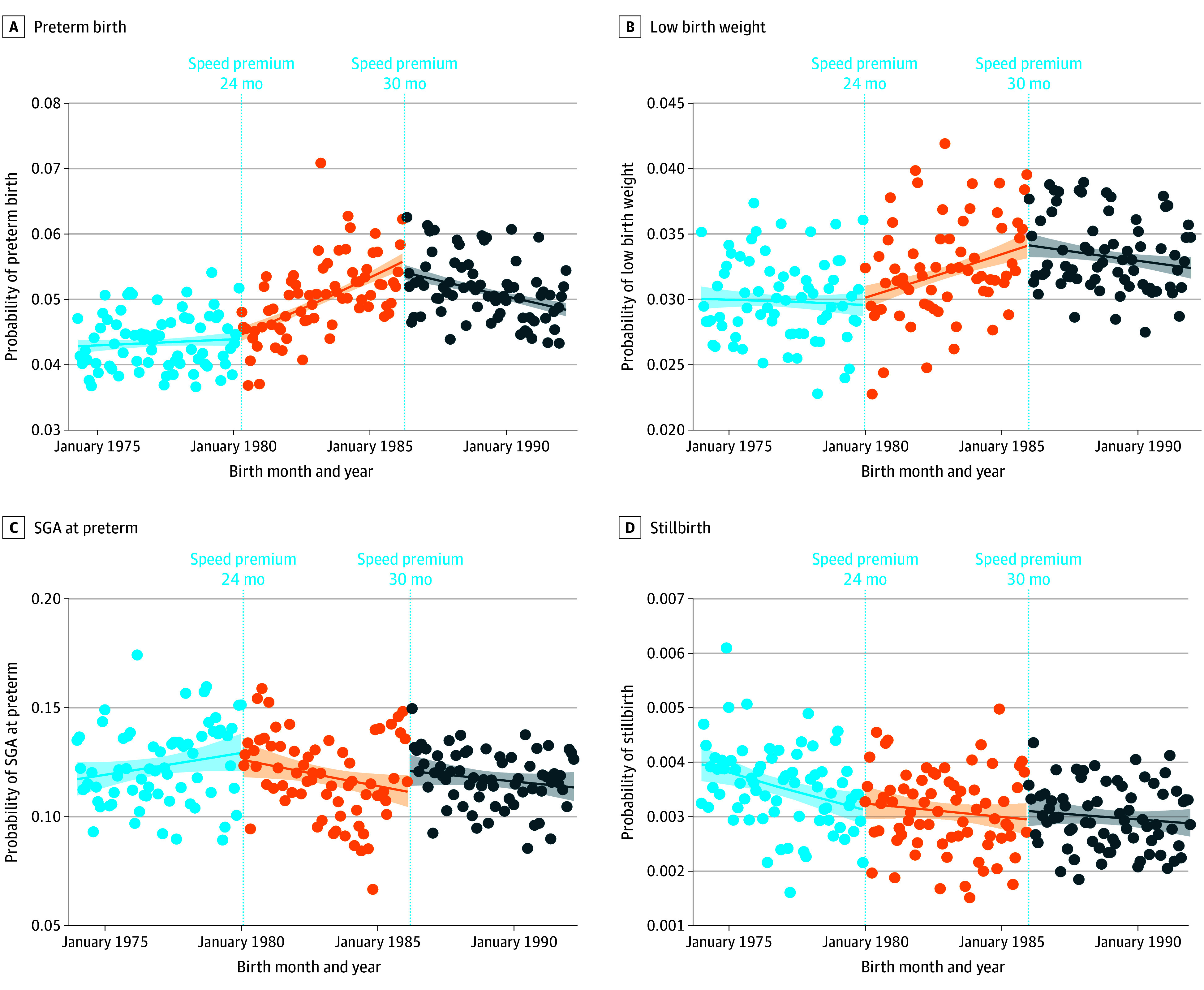
Monthly Probability of Perinatal Health Outcomes for Children Born From January 1, 1974, Through December 31, 1991 Data points indicate the observed probability; solid lines, estimated probability; and shaded areas, 95% CIs of the outcome from the individual-level interrupted time series analysis (eTable 1 in Supplement 1). The dashed vertical lines mark the introduction of the 24-month speed premium (January 1, 1980), and the later change to 30 months (January 1, 1986).

Results for preterm births showed a flat trend before 1980, increasing between January 1, 1980, and December 31, 1985, during the 24-month speed premium, and decreasing after January 1, 1986, when the policy was extended to 30 months (Figure 3A). More precisely, the 24-month reform was associated with a mean monthly increase in the odds of preterm births of 0.3% (OR, 1.0029; 95% CI, 1.002-1.004) compared with the trend before the reform (eTable 1 in the Supplement 1). In relative terms, this means that the odds of preterm births increased by 26.4% across the 6 years in which the 24-month speed premium was in place. After the 1986 reform extended the speed premium to 30-month intervals, the trend in preterm births reversed, decreasing on average by 0.5% per month (OR, 0.9951; 95% CI, 0.994-0.996) compared with 1980 to 1985, equivalent to an 11.1% decrease across the next 6 years. The results were unchanged when controlling for seasonality (eTable 2 in Supplement 1).

The odds of LBW similarly increased 0.2% (OR, 1.0021; 95% CI, 1.001-1.003) per month in 1980 through 1985 compared with baseline and decreased 0.3% (OR, 0.9975; 95% CI, 0.996-0.998) per month in the following period (Figure 3B; eTable 1 in Supplement 1). For the 24-month reform, this translated to 13.5% increased odds of LBW. Seasonally adjusted results remained unchanged (eTable 2 in Supplement 1). However, restricting the analysis to births at term indicated that the pattern for LBW was largely explained by preterm deliveries (eFigure 1 and eTable 3 in Supplement 1).

The speed premium reform was associated with decreased odds of SGA at preterm after 1980 (OR, 0.9965; 95% CI, 0.994-0.999) but not in 1986 (OR, 1.0009; 95% CI, 0.998-1.003) (Figure 3C and eTable 1 in Supplement 1). Stillbirths remained unchanged following both reforms (1980: OR, 1.0020 [95% CI, 0.999-1.005]; 1986: OR, 1.0002 [95% CI, 0.997-1.003]) (Figure 3D and eTable 1 in Supplement 1), with no further changes after controlling for seasonality (eTable 2 in Supplement 1).

Sensitivity analyses stratified by the mother’s origin showed that only native-born (1980: OR, 1.0030 [95% CI, 1.002-1.004]; 1986: OR, 0.9949 [95% CI, 0.994-0.996]) and other Nordic-born (1980: OR, 1.0037 [95% CI, 1.000-1.007]; 1986: OR, 0.9945 [95% CI, 0.991-0.998]) mothers experienced changed odds of preterm births around the speed premium reforms (eFigure 2 and eTable 4 in Supplement 1). Eastern European–born mothers exhibited trend changes similar to those of native-born mothers, albeit with wide confidence intervals (1980: OR, 1.0036 [95% CI, 0.998-1.009]; 1986: OR, 0.9944 [95% CI, 0.989-1.000]). Mothers of all other origins showed no changes in preterm births.

Models stratified by parity showed comparable results for odds of preterm between first- and higher-order births (eFigure 3 and eTable 5 in Supplement 1), suggesting that the policy may have been associated with perinatal health beyond shortened birth spacing alone. In fact, we found that the rate of births among women older than 40 years increased between 1980 and 1985, thereafter continuing to increase but at a slower rate (eFigure 4 in Supplement 1). We also found an increase in the rate of maternal diabetes, an age-related pregnancy complication, between 1980 and 1985 coinciding with the speed premium reform, which dropped significantly after the 1986 relaxation of the minimum birth-spacing criterion.

Sensitivity analyses using the last menstrual period alone to calculate gestational age (ie, without ultrasonography-based estimates) showed results comparable to those of the main analyses, suggesting that systemic changes in the approach to calculating gestational age during the reform periods did not bias the study findings (eFigure 5 and eTable 6 in Supplement 1). Finally, excluding births before 29 weeks’ gestation did not notably alter the results, suggesting that the increased odds of preterm birth after the 1980 speed premium introduction was not due to increased survival of children born extremely preterm (eFigure 6 and eTable 7 in Supplement 1). The results obtained after performing the analyses with aggregated data largely confirmed our findings, except for a lack of significant changes in SGA odds at preterm after the 1980 reform (eTable 8 in Supplement 1).

## Discussion

In this cross-sectional study, we analyzed the association of a Swedish family policy that aimed to protect parental leave benefits between births occurring at short intervals with perinatal health outcomes. We used an ITS analysis to evaluate trends of relevant perinatal outcomes following changes in the so-called speed premium policy in 1980 and 1986. The findings suggested that the introduction of income protection for 24-month birth intervals in 1980 was associated with increased odds of poor perinatal health, primarily preterm births and LBW, and its relaxation to 30-month intervals in 1986 was associated with a reversed trend in these risks.

By promoting the social and economic welfare of parents after birth, family policies have mainly been associated with positive health effects in the postpartum period, including reduced risks of infant mortality and improved developmental outcomes.^25^ This study used a unique approach by investigating the association of a family policy with pregnancy- and birth-related outcomes through anticipatory fertility behaviors used to maximize policy benefits. Our study results indicated that the speed premium appeared to affect birth outcomes only among mothers previously shown to adjust their fertility behaviors to the policy, namely, Swedish- and other Nordic-born mothers.^10^

Although we could not ascertain the degree to which the perinatal health changes were associated with specific fertility behaviors, both shortened birth spacing and increased maternal age may have played a role. Our observed association between short birth intervals and preterm births among multiparous mothers aligns with previous findings in Sweden.^5^ Previous evidence also showed that the reforms inadvertently increased the proportion of couples having children within the 24- and 30-month windows for income protection.^9,10^ Yet we also found elevated perinatal health risks among both first- and higher-order births, suggesting the presence of mechanisms other than short birth spacing alone (which could not plausibly impact first-order births). We theorized that the policy could additionally encourage childbearing at older ages, based on evidence of positive trends in maternal age during this period^26^ and the potentially greater advantages of income protection with higher earnings.^9^ Furthermore, birth spacing and maternal age could also be associated with each other through anticipatory mechanisms. Women aiming to have 2 or more children while postponing childbearing toward the end of their reproductive period may be influenced by the spacing eligibility of the speed premium, with longer spacing potentially encouraging a relatively earlier first birth. Although our data did not enable us to explore this hypothesis in greater detail, supplementary analyses showed that after the 1980 policy, there was an increase in the rate of women aged 40 years or older having children and in the rate of age-related pregnancy complications, trends which appeared to slow or reverse after the 1986 reform.

These changes in fertility behaviors likely resulted in perinatal health complications through a number of mechanisms. Research suggests that (very) short birth intervals may lead to heightened fetoplacental metabolic demand and depletion of maternal micronutrients (ie, iron and folate) resulting in adverse perinatal health.^4,27^ Advanced maternal age can impact perinatal outcomes by increasing risks of pregnancy-related complications in the mother, including hypertension and diabetes, thereby causing harm to the fetus.^19,28,29^ While we found increased odds of preterm birth and LBW associated with these preterm births, we found little to no evidence for an association between the speed premium and changes in the odds of pathologically growth-restricted fetuses (measured with SGA at preterm) or stillbirths over time. This lack of evidence is likely attributable to the severity of the latter 2 outcomes in a low-mortality context, such as Sweden. Furthermore, stillbirths appear to be associated with short birth spacing only through confounding maternal characteristics^30^ and with maternal age among nulliparous women.^31^

This study used total population medical birth data with relatively long follow-up before and after each policy to establish trends in the outcomes of interest. The availability of 2 reforms enabled us to design an ITS with multiple treatment periods. We not only observed changes in the relevant perinatal outcomes for 2 reforms, that is, both the introduction and partial relaxation of the speed premium, but also found that the changes were in opposite directions, discounting the possibility of other concurrent influences.^20,21^ Although the overall parental leave scheme was extended in 1982 and 1989, we are not aware of any concurrent reforms that could have affected the outcomes of interest. The results appeared to be robust to changed standards for calculating gestational age during the study period.^23^

To strengthen the validity of the results, the study would have benefited from a multiple-group comparison, based on distinctive treatment and control groups with comparable preintervention levels and trends in the outcomes of interest (ie, controlled before-after).^21^ Given that the policy was applicable to all individuals residing in Sweden, with a lack of valid historical comparisons, no control groups were available. However, the lack of conclusive evidence for perinatal health changes among immigrant mothers born outside of Nordic countries, a group previously shown to have no response to the policy,^10^ constituted a pseudocontrol condition supporting the association of the speed premium with the health outcomes observed among the exposed groups of native- and other Nordic-born mothers.

Our study contributes to discussions regarding the dark logic of social interventions, or how well-intended policies can cause harmful externalities or unintended adverse consequences.^32^ More specifically, given that evidence has long pointed to the negative consequences of short birth spacing,^33^ the speed premium reforms would have benefited from a Health in All Policies approach, as defined in the Helsinki Statement on Health in All Policies, decades ago (ie, by considering possible health externalities during the design process).^34^ Furthermore, the findings of this study may yet contribute to current debates regarding whether the reforms should be abolished in Sweden.^35^ To our knowledge, Sweden is the only country to provide income-protected parental leave benefits between births. Yet international policy makers can apply the findings of this study when developing their own family policies, especially in contexts aiming to make parenthood and work life more compatible and simultaneously increase fertility rates below replacement levels.^36^ Considering the costs of adverse perinatal outcomes, such as preterm births, which in the US can amount to more than $25 billion for 1 year of affected births,^37^ the incorporation of a Health in All Policies approach to family policy making could be of great societal value.

### Limitations

This study has limitations. One limitation is the lack of a comparable control group to conduct a controlled before-after analysis to further explain the association of underlying trends with the outcomes of interest.^21^ Changes in clinical practices of measuring gestational age may have also affected the results, although sensitivity analyses suggested that this was not the case. Finally, although we could not determine the exact extent to which the observed changes in birth outcomes were attributable to actual fertility changes (ie, birth spacing and maternal age at childbirth), additional analyses supported the role of both fertility changes in driving these results.

## Conclusions

The results of this cross-sectional study suggest that despite the economic protections of the speed premium policy for couples and especially for mothers in Sweden, its introduction was inadvertently associated with worse perinatal health outcomes, likely through changes in fertility behaviors. More research is needed to disentangle the precise mechanisms underlying the speed premium reforms and their associated perinatal health changes and to explore the long-term impacts of these changes for mothers and children alike.

## Notes

Supplement 1. eMethods. Interrupted time series methods with aggregated data **eTable 1.** Interrupted time series results for all perinatal health outcomes **eTable 2.** Interrupted time series results for all perinatal health outcomes, adjusted for seasonality **eTable 3.** Interrupted time series results for low birthweight at term **eTable 4.** Interrupted time series results for preterm births, by mother’s country or region of birth **eTable 5.** Interrupted time series results for preterm births, by parity **eTable 6.** Interrupted time series results for preterm births, using gestational age calculated with last menstrual period information only **eTable 7.** Interrupted time series results for preterm births, excluding extremely preterm births before week 29 **eTable 8.** Interrupted time series results for all perinatal health outcomes, using aggregated data **eFigure 1.** Interrupted time series model of low birthweight at term **eFigure 2.** Interrupted time series models of preterm births, by mother’s country or region of birth **eFigure 3.** Interrupted time series models of preterm births, by parity **eFigure 4.** Descriptive monthly rate of births at age 40 or above (Jan 1974-Dec 1991) and monthly rate of pregnancies with diabetes (Jan 1974-Dec 1989) **eFigure 5.** Interrupted time series model of preterm births, using gestational age calculated with last menstrual period information only **eFigure 6.** Interrupted time series model of preterm births, excluding extremely preterm births before week 29

Supplement 2.
Data Sharing Statement
