# Supplementary material for: Unintended Perinatal Health Consequences Associated With a Swedish Family Policy
Source: JAMA Pediatr. 2024 Apr 8;178(6):608–15. doi: 10.1001/jamapediatrics.2024.0378 (PMC11002779; doi:10.1001/jamapediatrics.2024.0378)
Supplement: Supplement 1. — eMethods. Interrupted time series methods with aggregated data eTable 1. Interrupted time series results for all perinatal health outcomes eTable 2. Interrupted time series results for all perinatal health outcomes, adjusted for seasonality eTable 3. Interrupted time series results for low birthweight at term eTable 4. Interrupted time series results for preterm births, by mother’s country or region of birth eTable 5. Interrupted time series results for preterm births, by parity eTable 6. Interrupted time series results for preterm births, using gestational age calculated with last menstrual period information only eTable 7. Interrupted time series results for preterm births, excluding extremely preterm births before week 29 eTable 8. Interrupted time series results for all perinatal health outcomes, using aggregated data eFigure 1. Interrupted time series model of low birthweight at term eFigure 2. Interrupted time series models of preterm births, by mother’s country or region of birth eFigure 3. Interrupted time series models of preterm births, by parity eFigure 4. Descriptive monthly rate of births at age 40 or above (Jan 1974-Dec 1991) and monthly rate of pregnancies with diabetes (Jan 1974-Dec 1989) eFigure 5. Interrupted time series model of preterm births, using gestational age calculated with last menstrual period information only eFigure 6. Interrupted time series model of preterm births, excluding extremely preterm births before week 29 [file jamapediatr-e240378-s001.pdf]

Supplementary Online Content

Debiasi E, Honkaniemi H, Aradhya S, Hjern A, Duvander AZ, Juárez SP. Unintended perinatal health consequences associated with a Swedish family policy. Published online April 1, 2024. *JAMA Pediatrics*. doi:10.1001/jamapediatrics.2024.0378

eMethods. Interrupted time series methods with aggregated data
eTable 1. Interrupted time series results for all perinatal health outcomes
eTable 2. Interrupted time series results for all perinatal health outcomes, adjusted for seasonality
eTable 3. Interrupted time series results for low birthweight at term
eTable 4. Interrupted time series results for preterm births, by mother's country or region of birth
eTable 5. Interrupted time series results for preterm births, by parity
eTable 6. Interrupted time series results for preterm births, using gestational age calculated with last menstrual period information only
eTable 7. Interrupted time series results for preterm births, excluding extremely preterm births before week 29
eTable 8. Interrupted time series results for all perinatal health outcomes, using aggregated data
eFigure 1. Interrupted time series model of low birthweight at term
eFigure 2. Interrupted time series models of preterm births, by mother's country or region of birth
eFigure 3. Interrupted time series models of preterm births, by parity
eFigure 4. Descriptive monthly rate of births at age 40 or above (Jan 1974-Dec 1991) and monthly rate of pregnancies with diabetes (Jan 1974-Dec 1989)
eFigure 5. Interrupted time series model of preterm births, using gestational age calculated with last menstrual period information only
eFigure 6. Interrupted time series model of preterm births, excluding extremely preterm births before week 29

This supplementary material has been provided by the authors to give readers additional information about their work.

eMethods. Interrupted time series methods with aggregated data

After aggregating data by birth month, we again employed an interrupted time series design with multiple treatment periods.[1,2] Based on the two reform dates (January 1980 and January 1986), the analyses were divided into three periods spanning a total of six years (January 1974–December 1979; January 1980–December 1985; and January 1986–December 1991) covering the three phases relative to changes in the speed premium policy as described in the paper. The data used and the analyzed outcomes are the same as in the main analysis.

We modeled the monthly rate of each outcome by fitting a linear ordinary least squares regression.[3] Thus, the regression coefficients reflect the average percentage-point change in the outcome rate from one month to the next. We used the Cumby-Huizinga test (actest command) to detect the appropriate autocorrelation structure per outcome.[4] Models were adjusted for seasonality when 12-month autocorrelation was detected by fitting Fourier terms (i.e., pairs of sine and cosine functions).[2] Confidence intervals based on lag-specific Newey-West standard errors were estimated to account for residual autocorrelation.

All statistical analyses were conducted using Stata Version 16.1. Results are reported in **eTable 8**.

References

1. Biglan A, Ary D, Wagenaar AC. The value of interrupted time-series experiments for community intervention research. *Prev Sci*. 2000;1(1):31.
2. Bernal JL, Cummins S, Gasparrini A. Interrupted time series regression for the evaluation of public health interventions: a tutorial. *Int J Epidemiol*. 2017;46(1):348-355.
3. Linden A. Conducting Interrupted Time-series Analysis for Single- and Multiple-group Comparisons. *SJ*. 2015;15(2):480-500.
4. Cumby RE, Huizinga J. Testing the Autocorrelation Structure of Disturbances in Ordinary Least Squares and Instrumental Variables Regressions. *Econometrica*. 1992;60(1):185-195.

© 2024 Debiasi E et al. *JAMA Pediatrics*.

eTable 1. Interrupted time series results for all perinatal health outcomes

	Preterm	Low birthweight	Small-for-gestational-age at preterm	Stillbirth
	Coefficient (95% CI)	Coefficient (95% CI)	Coefficient (95% CI)	Coefficient (95% CI)
Trend before 1980	1.00040 (0.99979 to 1.00101)	0.99974 (0.99901 to 1.00046)	1.00157 (0.99976 to 1.00338)	0.99669 (0.99461 to 0.99877)
Level change Jan 1980	1.01675 (0.98097 to 1.05383)	1.021517 (0.97811 to 1.06685)	0.96994 (0.87191 to 1.07898)	1.03662 (0.90931 to 1.18176)
Difference in trend 1974-1979 vs 1980-1985	1.00290 (1.00206 to 1.00374)	1.00205 (1.00103 to 1.00308)	0.99649 (0.99396 to 0.99901)	1.00200 (0.99890 to 1.00511)
Trend after 1980 (24-month *speed premium*)	1.00330 (1.00272 to 1.00388)	1.00179 (1.00107 to 1.00251)	0.99805 (0.99628 to 0.99982)	0.99868 (0.99640 to 1.00097)
Level change Jan 1986	0.96578 (0.93468 to 0.99791)	0.99935 (0.95945 to 1.04091)	1.09875 (0.99450 to 1.21393)	1.05330 (0.92184 to 1.20351)
Difference in trend 1980-1985 vs 1986-1991	0.99507 (0.99428 to 0.99585)	0.99746 (0.99648 to 0.99843)	1.00094 (0.99854 to 1.00334)	1.00024 (0.99710 to 1.00339)
Trend after 1986 (30-month *speed premium*)	0.99835 (0.99782 to 0.99888)	0.99924 (0.99859 to 0.99990)	0.99898 (0.99737 to 1.00060)	0.99892 (0.99677 to 1.00108)
Overall change Jan 1974-Dec 1979*	2.88% (-1.48% to 7.43%)	-1.83% (-6.79% to 3.32%)	11.78% (-1.69% to 27.07%)	-20.97% (-31.87% to -8.37%)
Overall change Jan 1980-Dec 1985*	26.35% (21.27% to 31.65%)	13.54% (7.89% to 19.48%)	-12.94% (-23.25% to -1.27%)	-8.95% (-22.59% to 7.13%)
Overall change Jan 1986-Dec 1991*	-11.06% (-14.35% to -7.65%)	-5.25% (-9.53% to -0.71%)	-6.99% (-17.05% to 4.35%)	-7.39% (-20.52% to 7.97%)
Observations	1,762,784	1,762,784	84,977	1,762,784

Data are odds ratios with 95% confidence intervals (CI) indicating the monthly percentage change in odds of the outcome of interest (see **Figure 3** for time series plots). *Cumulative change (and 95% CI) over the respective 6-year periods: e.g., between January 1980 and December 1985, the odds of preterm birth increased 0.3% every month, amounting to a 26.35% increase over the full 71 months (1.0033^71=1.2635).

© 2024 Debiasi E et al. *JAMA Pediatrics*.

eTable 2. Interrupted time series results for all perinatal health outcomes, adjusted for seasonality

	Preterm	Low birthweight	Small-for-gestational-age at preterm	Stillbirth
	Coefficient (95% CI)	Coefficient (95% CI)	Coefficient (95% CI)	Coefficient (95% CI)
Trend before 1980	1.00029 (0.99968 to 1.00090)	0.99957 (0.99884 to 1.00030)	1.00141 (0.99959 to 1.00323)	0.99663 (0.99454 to 0.99872)
Level change Jan 1980	1.02612 (0.98980 to 1.06378)	1.03516 (0.99090 to 1.08139)	0.97950 (0.87992 to 1.09036)	1.04121 (0.91253 to 1.18804)
Difference in trend 1974-1979 vs 1980-1985	1.00288 (1.00204 to 1.00373)	1.00204 (1.00102 to 1.00306)	0.99651 (0.99398 to 0.99903)	1.00200 (0.99890 to 1.00511)
Trend after 1980 (24-month *speed premium*)	1.00317 (1.00259 to 1.00376)	1.00161 (1.00088 to 1.00233)	0.99791 (0.99614 to 0.99969)	0.99862 (0.99633 to 1.00092)
Level change Jan 1986	0.97543 (0.94379 to 1.00812)	1.01342 (0.97268 to 1.05586)	1.110809 (1.00463 to 1.22821)	1.05845 (0.92558 to 1.21039)
Difference in trend 1980-1985 vs 1986-1991	0.99504 (0.99425 to 0.99582)	0.99743 (0.99646 to 0.99840)	1.00092 (0.99852 to 1.00333)	1.00022 (0.99708 to 1.00337)
Trend after 1986 (30-month *speed premium*)	0.99820 (0.99766 to 0.99873)	0.99903 (0.99837 to 0.99969)	0.99883 (0.99721 to 1.00046)	0.99884 (0.99668 to 1.00101)
Overall change Jan 1974-Dec 1979*	2.08% (-2.25% to 6.60%)	-3.01% (-7.91% to 2.15%)	10.52% (-2.87% to 25.73%)	-21.33% (-32.21% to -8.69%)
Overall change Jan 1980-Dec 1985*	25.20% (20.16% to 30.53%)	12.10% (6.44% to 17.97%)	-13.80% (-24.01% to -2.18%)	-9.34% (-22.98% to 6.75%)
Overall change Jan 1986-Dec 1991*	-12.01% (-15.32% to -8.63%)	-6.66% (-10.94% to -2.18%)	-7.98% (-17.99% to 3.32%)	-7.91% (-21.03% to 7.43%)
Observations	1,762,784	1,762,784	84,977	1,762,784

Data are odds ratios with 95% confidence intervals (CI) indicating the monthly percentage change in odds of the outcome of interest. Models are seasonally adjusted using monthly dummy variables. *Cumulative change (and 95% CI) over the respective 6-year periods: e.g., between January 1980 and December 1985, the odds of preterm birth increased 0.3% every month, amounting to a 25.20% increase over the full 71 months (1.0032^71=1.2520).

© 2024 Debiasi E et al. *JAMA Pediatrics*.

eTable 3. Interrupted time series results for low birthweight at term

	**Low birthweight**
	At term
	Coefficient (95% CI)
Trend before 1980	0.99869<br>(0.99733 to 1.00005)
Level change Jan 1980	1.08030<br>(0.99524 to 1.17264)
Difference in trend 1974-1979 vs 1980-1985	1.00182<br>(0.99988 to 1.00376)
Level change Jan 1986	0.97397<br>(0.89960 to 1.05447)
Difference in trend 1980-1985 vs 1986-1991	0.99898<br>(0.99710 to 1.00086)
Observations	1,677,807

Data are odds ratios with 95% confidence intervals (CI) indicating the monthly percentage change in odds of low birthweight (see **eFigure 1** for time series plot).

© 2024 Debiasi E et al. *JAMA Pediatrics*.

eTable 4. Interrupted time series results for preterm births, by mother's country or region of birth

	Preterm				
	Swedish	Nordic	Non-Western	Eastern European	Western
	Coefficient (95% CI)	Coefficient (95% CI)	Coefficient (95% CI)	Coefficient (95% CI)	Coefficient (95% CI)
Trend before 1980	1.00043 (0.99978 to 1.00108)	0.99909 (0.99685 to 1.00134)	0.99599 (0.99133 to 1.00067)	0.99941 (0.99558 to 1.00325)	1.00431 (0.99945 to 1.00920)
Level change Jan 1980	1.01903 (0.98047 to 1.05910)	1.01676 (0.88928 to 1.16252)	1.11714 (0.88783 to 1.40568)	1.05326 (0.84190 to 1.31768)	0.81876 (0.61112 to 1.09694)
Difference in trend 1974-1979 vs 1980-1985	1.00304 (1.00214 to 1.00395)	1.00368 (1.00046 to 1.00692)	1.00507 (0.99933 to 1.01083)	1.00358 (0.99823 to 1.00895)	0.99537 (0.98828 to 1.00252)
Trend after 1980 (24-month speed premium)	1.00347 (1.00285 to 1.00410)	1.00277 (1.00046 to 1.00509)	1.00103 (0.99775 to 1.00433)	1.00298 (0.99926 to 1.00671)	0.99966 (0.99442 to 1.00493)
Level change Jan 1986	0.96587 (0.93262 to 1.00032)	0.99008 (0.86063 to 1.13899)	0.94691 (0.80093 to 1.11950)	0.89187 (0.71620 to 1.11063)	1.13404 (0.83119 to 1.54724)
Difference in trend 1980-1985 vs 1986-1991	0.99492 (0.99408 to 0.99577)	0.99454 (0.99120 to 0.99789)	0.99775 (0.99371 to 1.00180)	0.99439 (0.98919 to 0.99961)	0.99858 (0.99124 to 1.00597)
Trend after 1986 (30-month speed premium)	0.99838 (0.99781 to 0.99895)	0.99730 (0.99485 to 0.99975)	0.99878 (0.99641 to 1.00115)	0.99735 (0.99367 to 1.00104)	0.99824 (0.99308 to 1.00342)
Observations	1,551,501	101,282	56,053	32,006	21,942

Data are odds ratios with 95% confidence intervals (CI) indicating the monthly percentage change in odds of preterm birth by mothers' country or region of birth (see **eFigure 2** for time series plots).

© 2024 Debiasi E et al. *JAMA Pediatrics*.

eTable 5. Interrupted time series results for preterm births, by parity

	Preterm	
	Parity = 1	Parity > 1
	Coefficient (95% CI)	Coefficient (95% CI)
Trend before 1980	1.00114<br>(1.00026 to 1.00203)	0.99994<br>(0.99910 to 1.00077)
Level change Jan 1980	1.02474<br>(0.97246 to 1.07982)	1.00894<br>(0.96056 to 1.05975)
Difference in trend 1974-1979 vs 1980-1985	1.00253<br>(1.00130 to 1.00375)	1.00310<br>(1.00194 to 1.00426)
Level change Jan 1986	0.99064<br>(0.94506 to 1.03843)	0.93393<br>(0.89229 to 0.97751)
Difference in trend 1980-1985 vs 1986-1991	0.99487<br>(0.99375 to 0.99600)	0.99506<br>(0.99397 to 0.99616)
Observations	740,773	1,022,011

Data are odds ratios with 95% confidence intervals (CI) indicating the monthly percentage change in odds of preterm birth by parity (see **eFigure 3** for time series plots).

eTable 6. Interrupted time series results for preterm births, using gestational age calculated with last menstrual period information only

	Preterm
	Last menstrual period
	Coefficient (95% CI)
Trend before 1980	1.00037<br>(0.99970 to 1.00104)
Level-change January 1980	1.01346<br>(0.97336 to 1.05520)
Difference in trend 1974-1979 vs 1980-1985	1.00261<br>(1.00164 to 1.00359)
Level-change January 1986	0.95660<br>(0.91513 to 0.99994)
Difference in trend 1980-1985 vs 1986-1991	0.99516<br>(0.99404 to 0.99628)
Observations	1,229,812

Data are odds ratios with 95% confidence intervals (CI) indicating the monthly percentage change in odds of preterm birth (see **eFigure 5** for time series plot).

© 2024 Debiasi E et al. *JAMA Pediatrics*.

eTable 7. Interrupted time series results for preterm births, excluding extremely preterm births before week 29

	Preterm
	Excluding gestational age <29 weeks
	Coefficient (95% CI)
Trend before 1980	1.00059<br>(0.99997 to 1.00121)
Level change Jan 1980	1.01648<br>(0.98005 to 1.05426)
Difference in trend 1974-1979 vs 1980-1985	1.00264<br>(1.00178 to 1.00350)
Level change Jan 1986	0.96466<br>(0.93296 to 0.99743)
Difference in trend 1980-1985 vs 1986-1991	0.99492<br>(0.99412 to 0.99572)
Observations	1,759,055

Data are odds ratios with 95% confidence intervals (CI) indicating the monthly percentage change in odds of preterm birth (see **eFigure 6** for time series plot).

© 2024 Debiasi E et al. *JAMA Pediatrics*.

eTable 8. Interrupted time series results for all perinatal health outcomes, using aggregated data

	Preterm	Low birthweight	Small-for-gestational-age at preterm	Stillbirth
	Coefficient (95% CI)	Coefficient (95% CI)	Coefficient (95% CI)	Coefficient (95% CI)
Trend before 1980	0.0012<br>(-0.0018 to 0.0043)	-0.0012<br>(-0.0034 to 0.00097)	0.018<br>(-0.0014 to 0.037)	-0.0012<br>(-0.0019 to -0.00047)
Level change Jan 1980	0.091<br>(-0.086 to 0.27)	0.10<br>(-0.038 to 0.24)	-0.33<br>(-1.72 to 1.07)	0.014<br>(-0.032 to 0.059)
Difference in trend 1974-1979 vs 1980-1985	0.014<br>(0.0088 to 0.019)	0.0061<br>(0.0029 to 0.0093)	-0.039<br>(-0.080 to 0.0010)	0.00081<br>(-0.00033 to 0.0020)
Trend after 1980 (24-month *speed premium*)	0.015<br>(0.011 to 0.020)	0.0049<br>(0.0025 to 0.0073)	-0.022<br>(-0.057 to 0.014)	-0.00039<br>(-0.0013 to 0.00038)
Level change Jan 1986	-0.14<br>(-0.39 to 0.11)	0.042<br>(-0.086 to 0.17)	1.03<br>(-0.77 to 2.83)	0.015<br>(-0.031 to 0.062)
Difference in trend 1980-1985 vs 1986-1991	-0.024<br>(-0.029 to -0.019)	-0.0081<br>(-0.011 to -0.0050)	0.010<br>(-0.030 to 0.051)	0.000050<br>(-0.0010 to 0.0011)
Trend after 1986 (30-month *speed premium*)	-0.0085<br>(-0.011 to -0.0057)	-0.0032<br>(-0.0052 to -0.0011)	-0.011<br>(-0.027 to 0.0044)	-0.00034<br>(-0.0010 to 0.00030)
Overall change Jan 1974-Dec 1979*	2.05%<br>(-3.12% to 7.21%)	-2.81%<br>(-7.88% to 2.26%)	10.83%<br>(-1.51% to 23.16%)	-21.64%<br>(-34.82% to -8.45%)
Overall change Jan 1980-Dec 1985*	24.16%<br>(19.45% to 28.88%)	11.40%<br>(5.91% to 16.88%)	-12.10%<br>(-24.59% to 0.39%)	-8.60%<br>(-27.75% to 10.55%)
Overall change Jan 1986-Dec 1991*	-11.12%<br>(-14.79% to -7.44%)	-6.51%<br>(-10.73% to -2.29%)	-6.60%<br>(-15.01% to 1.80%)	-7.81%<br>(-22.42% to 6.80%)
Observations	216	216	216	216

Data are β-coefficients with 95% Confidence Intervals (CI) indicating the absolute monthly percentage-point change in the outcome of interest. Models are seasonally adjusted using Fourier terms and consider lag-specific Newey-West standard errors in presence of residual autocorrelation. *Total relative change (and 95% CI) over the respective 6-year periods; e.g., the rate of preterm births was 4.49% in January 1980 and 5.57% in December 1985, amounting to a 24.16% increase during the full 1980-1985 period (4.49*1.2416=5.57).

© 2024 Debiasi E et al. *JAMA Pediatrics*.

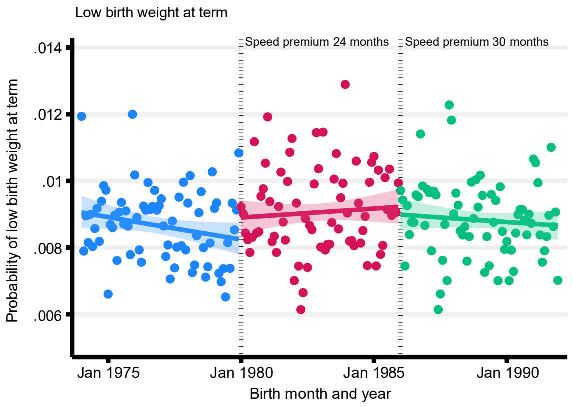

eFigure 1. Interrupted time series model of low birthweight at term

Dots indicate the observed probability, solid lines the estimated probability, and shaded areas the 95% confidence intervals (CI) of the outcome from the individual-level interrupted time series analysis (see **eTable 3**). The dashed vertical lines mark the introduction of the 24-month *speed premium* in January 1980 and the later change to 30 months in January 1986.

© 2024 Debiasi E et al. *JAMA Pediatrics*.

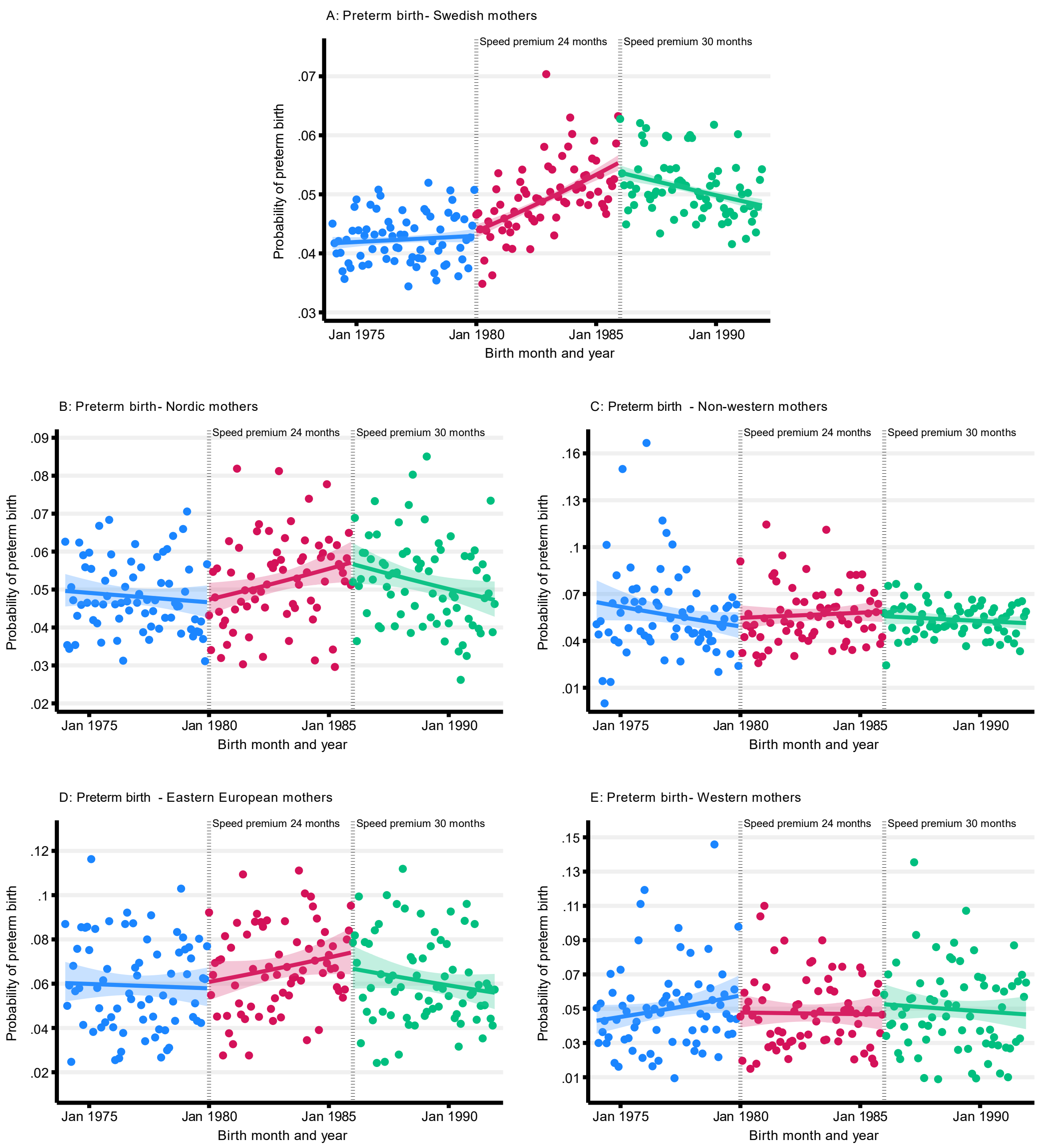

eFigure 2. Interrupted time series models of preterm births, by mother's country or region of birth

Panel A: Preterm birth among Swedish mothers; B: Preterm birth among Nordic mothers; C: Preterm birth among Non-Western mothers; D: Preterm birth among Eastern European mothers; E: Preterm birth among Western mothers. Dots indicate the observed probability, solid lines the estimated probability, and shaded areas the 95% confidence intervals (CI) of the outcome from the individual-level interrupted time series analysis (see **eTable 4**). The dashed vertical lines mark the introduction of the 24-month *speed premium* in January 1980 and the later change to 30 months in January 1986.

© 2024 Debiasi E et al. *JAMA Pediatrics*.

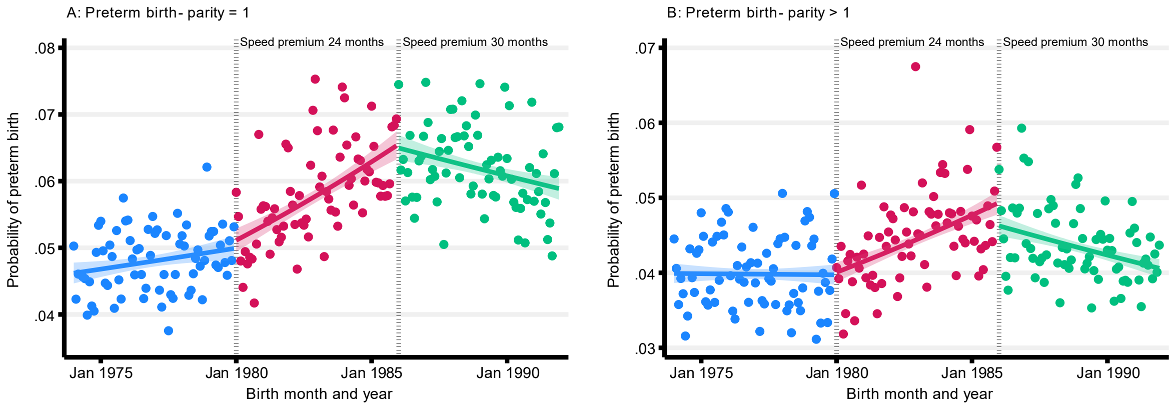

eFigure 3. Interrupted time series models of preterm births, by parity

Panel A: Preterm birth with Parity 1; B: Preterm birth with Parity>1. Dots indicate the observed probability, solid lines represent the estimated probability, and shaded areas represent the 95% confidence intervals (CI) of the outcome from the individual-level interrupted time series analysis (see **eTable 5**). The dashed vertical lines mark the introduction of the 24-month *speed premium* in January 1980 and the later change to 30 months in January 1986.

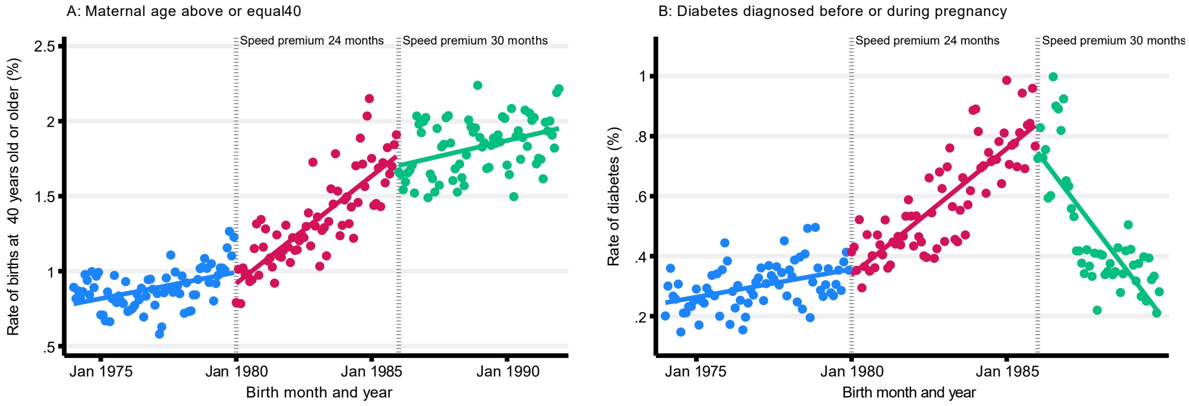

eFigure 4. Descriptive monthly rate of births at age 40 or above (Jan 1974-Dec 1991) and monthly rate of pregnancies with diabetes (Jan 1974-Dec 1989)

Panel A: Births to mothers age 40 or above (Jan 1974-Dec 1991); B: Births to mothers diagnosed with maternal diabetes before or during pregnancy (Jan 1974-Dec 1989). Dots indicate the observed rates while the solid black line represents the fitted linear trend. The dashed vertical lines mark the introduction of the 24-month *speed premium* in January 1980 and the later change to 30 months in January 1986.

© 2024 Debiasi E et al. *JAMA Pediatrics*.

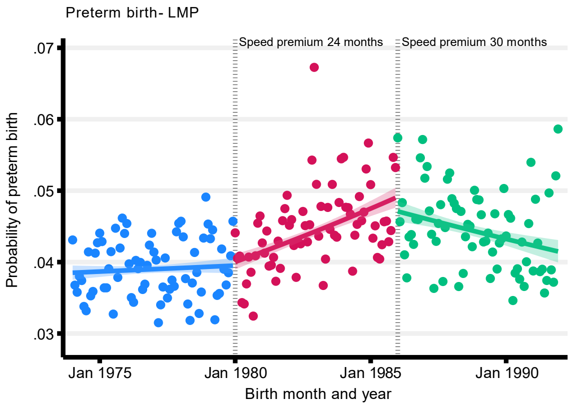

eFigure 5. Interrupted time series model of preterm births, using gestational age calculated with last menstrual period information only

Dots indicate the observed probability, solid lines the estimated probability, and shaded areas the 95% confidence intervals (CI) of the outcome from the individual-level interrupted time series analysis (see **eTable 6**). The dashed vertical lines mark the introduction of the 24-month *speed premium* in January 1980 and the later change to 30 months in January 1986.

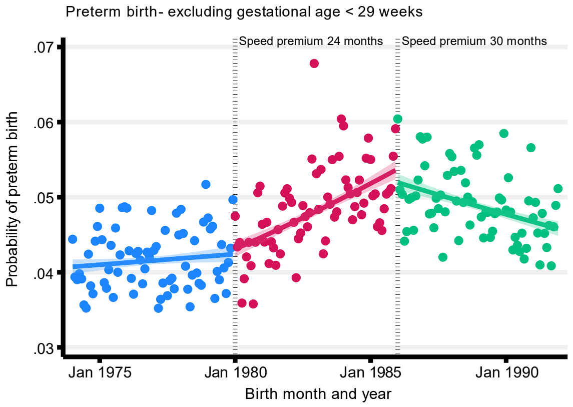

eFigure 6. Interrupted time series model of preterm births, excluding extremely preterm births before week 29

Dots indicate the observed probability, solid lines the estimated probability, and shaded areas the 95% confidence intervals (CI) of the outcome from the individual-level interrupted time series analysis (see **eTable 7**). The dashed vertical lines mark the introduction of the 24-month *speed premium* in January 1980 and the later change to 30 months in January 1986.

© 2024 Debiasi E et al. *JAMA Pediatrics*.
