# Supplementary material for: Unintended Perinatal Health Consequences Associated With a Swedish Family Policy
Source: JAMA Pediatr. 2024 Apr 8;178(6):608–15. doi: 10.1001/jamapediatrics.2024.0378 (PMC11002779; doi:10.1001/jamapediatrics.2024.0378)
Supplement: Supplement 2. — Data Sharing Statement [file jamapediatr-e240378-s002.pdf]

Data Sharing Statement

Debiasi. Unintended Perinatal Health Consequences Associated With a Swedish Family Policy. *JAMA Pediatr*. Published April 08, 2024. doi:10.1001/jamapediatrics.2024.0378

Data
Data available: No

Additional Information
Explanation for why data not available: The data are available from Statistics Sweden under license for the current study, and are not publicly available.
